# A Novel Method for the Separation of Overlapping Pollen Species for Automated Detection and Classification

**DOI:** 10.1155/2016/5689346

**Published:** 2016-03-10

**Authors:** Santiago Tello-Mijares, Francisco Flores

**Affiliations:** ^1^Departamento de Posgrado, Instituto Tecnológico Superior de Lerdo, Tecnológico 1555, Placido Domingo, 35150 Lerdo, DG, Mexico; ^2^Departamento de Posgrado, Instituto Tecnológico de la Laguna, Boulevard Revolución, Centro, 27000 Torreón, CO, Mexico

## Abstract

The identification of pollen in an automated way will accelerate different tasks and applications of palynology to aid in, among others, climate change studies, medical allergies calendar, and forensic science. The aim of this paper is to develop a system that automatically captures a hundred microscopic images of pollen and classifies them into the 12 different species from Lagunera Region, Mexico. Many times, the pollen is overlapping on the microscopic images, which increases the difficulty for its automated identification and classification. This paper focuses on a method to segment the overlapping pollen. First, the proposed method segments the overlapping pollen. Second, the method separates the pollen based on the mean shift process (100% segmentation) and erosion by H-minima based on the Fibonacci series. Thus, pollen is characterized by its shape, color, and texture for training and evaluating the performance of three classification techniques: random tree forest, multilayer perceptron, and Bayes net. Using the newly developed system, we obtained segmentation results of 100% and classification on top of 96.2% and 96.1% in recall and precision using multilayer perceptron in twofold cross validation.

## 1. Introduction

In allergic rhinitis and asthmatic exacerbations, major allergens are dust mites, followed by pollen. Martínez Ordaz et al. [1] found a significant correlation between the concentration of environmental pollen and the frequency of asthmatic exacerbations in the Lagunera Region in north-central Mexico, with Pearson's *r* of 0.63 and a coefficient of determination *r* of 0.39 (*p* < 0.01). This is due to the pollution of a metropolitan area of 1.5 million inhabitants and an arid climate where rain is not often present to cleanse the air of pollutants. Additionally, Campos et al. [2] observed a significant relationship between the concentration of Chenopodiaceae and Amaranthaceae pollen particles and a peak expiratory flow in the same region.

**Figure 1:**
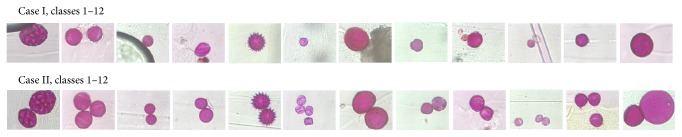
Dataset of examples of pollen species images classified according to Table 1. Case I, classes 1–12, and Case II, classes 1–12.

Over the past decade, interest has increased in automatic systems to identify pollen, which can be helpful for palynologists and medical specialists. Such a system could help make climate change studies, medical allergies calendar, and forensic science easier. Techniques using such an automated system could help to reduce errors of palynologist scanning. In developing such an automated system, a new challenge is presented: the segmentation of overlapped pollen for species classification. This study is part of a project that aims to develop an automated system that captures hundreds of microscopic images of pollen to select and classify these images for calendaring. In this paper, we focus on the first stage of system analysis that aims to segment and classify 12 species of pollen into 12 palynological categories (Table 1). In real samples, pollen grains can be found segregated or overlapped (Figure 1 Case I or Case II, resp.), which increases the detection difficulty. Also, in the background of the images we found Vaseline and other unwanted materials. Pollen grains are 10–60 *μ*m diameter structures that contain reproduction matter. The pollen is covered with two membranes (exine and intine). The exine membrane can have porous and/or elongated grooves, or none, to permit transfer of genetic material in the pollination process [3]. Thus, we proposed a dataset composed of 12 pollen species divided into 12 palynological classes, as can be seen in Table 1 and Figure 1, in order to apply a segmentation method for pollen detection and evaluation of species by three different classification methods.

Besides quality of detection and expressivity of the used visual descriptors, the estimated accuracy rate for a recognition system is directly related to the number of classes in the dataset and the statistical significance of the proposed evaluation schemes. Several proposals to detect pollen grains can be found in the literature (Rodriguez-Damian et al. [3]; Ranzato et al. [4]; Mitsumoto et al. [5]; Kaya et al. [6]; Dell'Anna et al. [7]), and we have compared them to the method shown in this paper. According to Rodriguez-Damian et al. [3] and Mitsumoto et al. [5], pollen grains have a circular shape (assumed shape). Since the pollen species they analyzed presented this circular shape, it was therefore a reasonable restriction. Ranzato et al. [4] presented work using a generic filtering approach. In relation to the characterization of detected grains, different visual descriptors are also proposed. Rodriguez-Damian et al. [3] computed shape and texture descriptors over the segmented grains, but only three pollen species are under consideration, and the same data are used for training and testing. Ranzato et al. [4] used local texture descriptors and worked with twelve classes separated into 10 and 100 randomly generated experiments, with 90% of the set used for training and 10% for testing. Mitsumoto et al. [5] applied a very simple descriptor (pollen size and the ratio of the blue to red pollen autofluorescence spectra). They concluded that, using their descriptors, two pollen species can be separated, although they do not present accuracy rates or evaluation schemes. Kaya et al. [6] used 11 different microscopic and morphological characteristic features for a rough set-based expert system for the classifications of twenty different pollen grains from the Onopordum family, obtaining a 90.625% (145/160) rate of success. Finally, Dell'Anna et al. [7] used spectral reflectance from Fourier transform infrared (FT-IR) microspectroscopy with unsupervised (hierarchical cluster analysis, HCA) and supervised (k-NN neighbors classifier, k-NN) learning methods to discriminate and automatically classify pollen grains from 11 different allergy-relevant species belonging to different 7 families (5 single pollen grains per species). The k-NN classifier they built got an overall accuracy of 84% and, for nine out of the 11 considered plant species, the obtained accuracy was greater than or equal to 80%.

In this work, we propose a generic approach for grain detection based on mean shift segmentation and applying Otsu's method, morphological erosion, and dilatation using the H-minima method based on Fibonacci series and gradient vector flow snake (GFVS) when the grains overlap. On the other hand, as opposed to Rodriguez-Damian et al. [3] and Mitsumoto et al. [5], the shape of the grains does not have to be circular Also, a significant evaluation of current visual descriptors is performed, applying to three classification techniques (Tree Random Forest, Multilayer Perceptron, and Bayes Net) using three *s*-fold cross-validation schemes (*s* = 10, *s* = 5, and *s* = 2): 2-fold setup (2 iterations, 50% training, and 50% test), 5-fold (5 iterations, 80% training, and 20% test), and 10-fold (10 iterations, 90% training, and 10% test). Thus, we propose a novel approach that automates imaging and classification of pollen.

## 2. Materials and Methods

**Figure 2:**
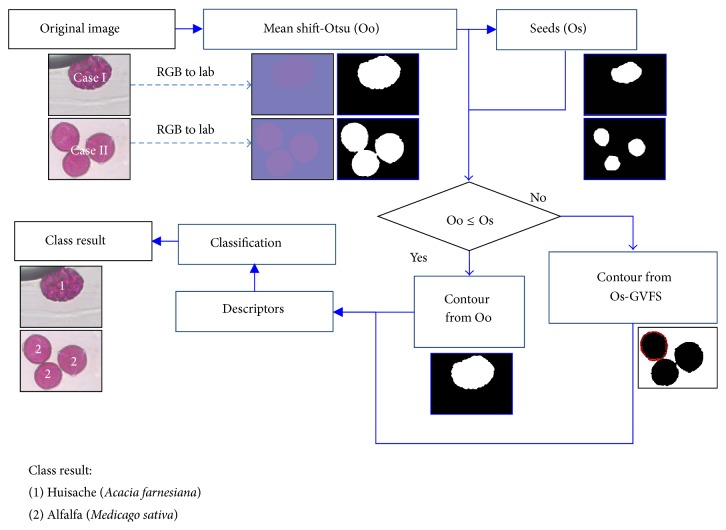
Overall method description.

Figure 2 illustrates the stages of the proposed segmentation algorithm and classification techniques, which are detailed in the following subsections. First, we explain the slide preparation and image acquisition and, later, the proposed system. The proposed system is accomplished in four stages: image preprocessing (RGB to lab), pollen image segmentation (mean shift and Otsu's method, followed by the H-minimum process based on the Fibonacci series to identify the overlapping pollen and finally applying gradient vector flow snake (GVFS), feature extraction (shape, color, and texture), and classification (into 12 pollen species).

### 2.1. Slide Preparation and Image Acquisition

Pollen grains were acquired by air sampling and sediment technique; in our case, we used a pollen collector model Hirst, commercial brand Burkard (Burkard Mfg. Co. Ltd., Rickmansworth, Hertfordshire, England), and the pollen slides were prepared according to the Wodehouse technique for light microscope [8]:

- A small amount of pollen, about as much as can be picked up on the flat end of toothpick, is placed on the center of microscopic slide and a drop of alcohol added and allowed partly to evaporate (one to four drops were added).
- The alcohol spreads out as it evaporates and leaves the oily and resinous substances of the pollen deposited in ring form around the specimen.
- The oily ring is wiped off with the help of cotton moistened with alcohol and before the specimen dried completely, a small drop of hot melted methyl green glycerin jelly was added, and the pollen stirred in with the needle and is evenly distributed. During the process the jelly is kept hot by passing the slide over a small flame.
- Cover glass (#1) was then placed over the specimen and the slide gently heated.

For the image acquisition, an Axioskop 40 microscope with an AxioCam MR Series digital camera (Carl Zeiss Microscopy, LLC, EUA) that has a sensor of 8.9 × 6.7 mm (2/3′′), with 1.4 megapixels and 12 bits of digitalization was used. Available exposition time goes from 1 millisecond to 20 seconds and is capable of video capture at 38 frames per second with a resolution of 276 × 208 pixels.

### 2.2. Preprocessing

Input RGB pollen images (see Figure 2) are converted to lab color space. The perceptual linearity makes it more suitable for distance-based pollen region segmentation, which is the following step.

### 2.3. Segmentation

**Figure 3:**
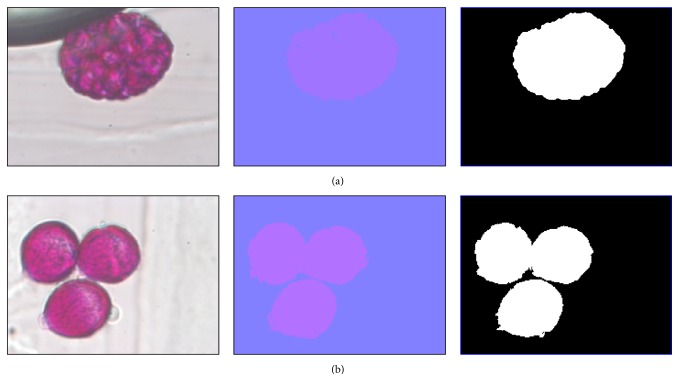
Segmentation of the pollen images. Shown left to right are original image, mean shift, and Otsu's method. (a) Case I and (b) Case II.

Mean shift's segmentation refers to the process of partitioning an image into multiple segments [9]. We propose the use of the mean shift algorithm [10] to obtain the segmentation of the pollen region from the microscopic images. Mean shift is a nonparametric technique for analyzing multimodal data, with multiple applications in pattern analysis [11], including its use for image segmentation. We start from the observation that the pollen in a region, either individual (Figure 3(a)) or overlapping (Figure 3(b)), is characterized by similar color values (always similar to purple, Figure 3), unlike the area around the pollen (such as the background, Vaseline, and unwanted material). We characterize every pixel of the image by a vector *x*_*i*_ = [*a*, *b*], with [*L*, *a*, *b*] being its color. We then run the mean shift algorithm over this 2-dimensional distribution with a bandwidth value *h* = 25, selected so that cell pollen is segmented into two or three regions, which is required for the later use of Otsu's [12] binary process to be effective and to only have two regions. Once the pollen region is segmented, the next step is to separate the overlapping pollen using an effective erosion method inspired in H-minimal and based on Fibonacci series to identify whether it belongs to Case I or Case II.

### 2.4. H-Minima Based on the Fibonacci Series

**Figure 4:**
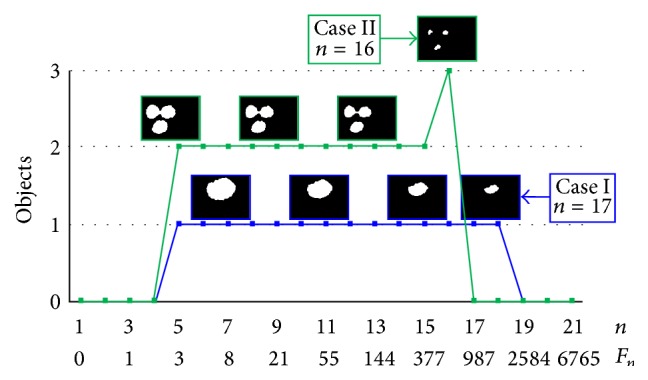
H-minima based erosion by the Fibonacci series for binary seeds image.

After segmentation of the pollen region, the next step is to separate the overlapping pollen using an effective proposed method of erosion inspired in H-minimal and based on the Fibonacci series to identify whether the region pollen belongs to Case I or Case II. Morphological erosion using a disk shaped structural element can be used to identify the type of region (Case I or II). This erosion can be performed *n* times from *n* = 1 : 21, defining the radius *R* of the disk using the Fibonacci series *Fn* = 0,1, 3 ⋯ 6765, as seen in Figure 4. The pollen region becomes smaller as it is eroded until it disappears altogether. When the pollen region belongs to Case I, it is an object or a number of objects (separate pollen grains delineated in blue in Figure 4) both at the start and near the very end of the erosion process. On the other hand, when the region belongs to Case II, as in the green line in Figure 4, at the beginning there are two objects that become three objects just before they disappear (overlapping pollen grains). Because of the disk structure, the pollen region can be separated because the pollen grains have an elliptical or a circular shape. Under this condition, when the final count of binary objects is different from the initial count, it belongs to Case II, and when the initial and final count is the same, it belongs to Case I. Figure 2 shows the results of applying this morphological operation. After an image is classified as belonging to Case II, a morphological dilation is applied until the number of elements (separate pollen grains) remains the same. These objects will be the initial seeds for the GVFS application.

### 2.5. Pollen Separation by Gradient Vector Flow Snakes

**Figure 5:**
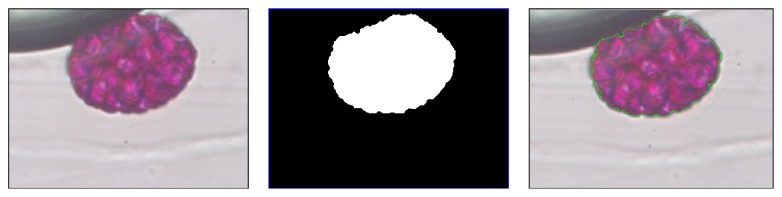
Feature extraction, Case I.

Once an image is identified as Case I, we proceed to extract the features based on the initial segmented object (Figure 5).

**Figure 6:**
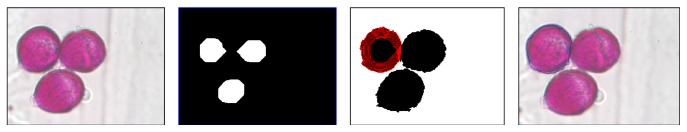
Feature extraction of mean GVFS, Case II.

For Case II, we can observe the numerical difference between objects, as seen in Figure 4. Therefore, to recover the pollen separated from the pollen region, we used dilated objects (Figure 6). These objects can work like seeds and the gradient vector flow snakes (GVFS) may be applied.

Traditional snakes are curves (*x*(*s*) = [*x*(*s*), *y*(*s*)], *s* ∈ [0,1]) defined within the domain of an image, and it can move itself and allow them to move under the influence of internal forces coming from within the curve itself and external forces computed from the image data, as first introduced by Kass et al. [13]. The GVFS improves the capture range of the contours obtained by the binary image. Xu and Prince [14] propose an improved snake to obtain better performance for image segmentations (Figure 6). The formulation of a GVFS is valid for gray images as well as binary images; however, we used binary images, as seen in Figure 6. To compute GVFS, an edge-map function is first calculated using a Gaussian function. An edge-map function and an approximation of its gradient are then given. The GVFS is computed to guide the deformation of the snake at the boundary edges.

### 2.6. Feature Extraction

This part aims to characterize pollen grains separated with a feature vector that helps classify 12 species. These pollen regions are then mapped on the *L* ^*∗*^ *a* ^*∗*^ *b* ^*∗*^ color model image for feature extraction. Selected features can be grouped into three categories that are summarized in Table 2.

#### 2.6.1. Shape Descriptors

Length and width of the bounding box (*MA* = length and *mA* = width) are obtained from the separated pollen grains. The area (*A*) of the grain is determined by counting the number of pixels contained within the border. Perimeter (*P*) is the length of the border. The obtained regions *A* and *P* can be used as descriptors because the size of the pollen grain is a palynological parameter of interest. Roundness (*R*) is defined as the multiplication of 4*π* and *A* over squared *P*. If *R* = 1, then the object is circular. Compactness (*C*) is defined as the result of *A* over *P*. 4*π*

#### 2.6.2. First-Order Texture Descriptors

One way to discriminate between different textures is to compare *L* ^*∗*^, *a* ^*∗*^, and *b* ^*∗*^ levels using first-order statistics. First-order statistics are calculated based on the probability of observing a particular pixel value at a randomly chosen location in the image. They depend only on individual pixel values and not on the interaction of neighboring pixel values. Average (*μ*) is the mean of the sum of all intensity values in the image. Median (*m*) represents the value of the central variable position in the dataset of sorted pixels. Variance (*σ* ^2^) is a dispersion measure defined as the squared deviation of the variable with respect to its mean. Standard deviation (*σ*) is a measure of centralization or dispersion variable. Entropy (*S*) of the object in the image is a measure of content information.

#### 2.6.3. Second-Order Texture Descriptors

Haralick's gray level cooccurrence matrices [15] have been used very successfully for biomedical image classification [16, 17]. Out of 14 features outlined, we considered first 4 texture features suitable for our experiment. We propose to use the cooccurrence matrix for the whole *L* ^*∗*^ *a* ^*∗*^ *b* ^*∗*^ color model. Contrast descriptor (CM) is a measure of local variation in the image. It is a high value when the region within the range of the window has a high contrast. Correlation (*r*) of the texture measures the relationship between the different intensities of colors. Mathematically, the correlation increases when the variance is low, meaning that the matrix elements are not far from the main diagonal. Energy (*e*) is the sum of the squared elements in the matrix of cooccurrence of gray level, also known as the uniformity or the second element of the angular momentum. Local homogeneity (HL) provides information on local regularity of the texture. When the elements of the cooccurrence matrix are closer to the main diagonal, the value of the local homogeneity is higher.

### 2.7. Classification

The main contribution of this work is the proposed segmentation and characterization method for the classification of pollen. In order to classify segmented pollen into 12 pollen species and obtain final classification results, we have explored the use of three different classification approaches implemented in Weka (Waikato Environment for Knowledge Analysis) [18, 19]: random tree forests [20] (RTF), a multilayer perceptron (MLP) and a Bayesian network (BN). Experimental results have been obtained for these three classification techniques (see confusion matrixes in Table 3 and Section 3).

## 3. Results and Discussion

For pollen classification in this work, the quantitative and qualitative results evaluation of three different classification techniques, namely, random tree forest (RTF, Table 3), multilayer perceptron (MLP, Table 4), and Bayes net (BN, Table 5), was measured using three different *s*-fold schemes (*s* = 2, *s* = 5, and *s* = 10) in cross validation. The first choice was the 2-fold cross validation, where the dataset was divided into two equal parts (50% training set, 50% test set) and the other two were selected to be 5-fold (80% training set, 20% test set) and 10-fold (90% training set, 10% test set). Tables 3 to 5 show the confusion matrixes results for the twelve pollen classes (*a* = 1, *b* = 2,…, *l* = 12, according to Table 1) of the classification techniques (RTF, MLP, and BN). A confusion matrix contains information about actual and predicted classifications done by a classification technique [21].

### 3.1. Dataset Description

The dataset and associated ground-truth is composed of 389 images from 12 different pollen species (Table 1). The images are formed using greater magnification and resolution (1388 × 1040 pixels). The experiments and resulting images are 278 × 208 × 3 pixels, as the application is in video format. To obtain the ground-truth, the contours of the pollen grains were manually identified by an expert palynologist. The Supplementary Materials for download contain the entire pollen images database (classes 1–12), the ground-truth (as binary bmp images), results images segmentation (as Matlab figures), Excel descriptors of the features of every pollen, Weka features and associated class for experiment (as ARFF Data File), and segmentation method (as Matlab interface) (see Supplementary Material available online at http://dx.doi.org/10.1155/2016/5689346).

### 3.2. Quality Indicators for Pollen Classification

In order to quantitatively assess the pollen classification results and the performance of the RTF, MLP, and BN techniques, several quality indicators were obtained. They were divided into final or external quality indicators that evaluate the final classification results and are useful for external comparison with other works or internal quality indicators that are useful for evaluating the internal behavior of the proposed classification options. For external indicators, let P be the number of pollen substances in the dataset, and let TP, FP, and FN be the number of true positives, false positives, and false negatives, respectively. We then defined TP rate, recall, or sensitivity as TPR = TP/P = TP/TP + FN and precision or positive predictive value as PPV = TP/TP + FP. As the proposed algorithm first selects the pollen that is then characterized and separated into 12 pollen classes, we can further evaluate the classification performance of the three selected classification schemes via the internal indicators. Here, let N be the number of nonpollen candidates resulting from the application of the proposed method to the complete dataset and let TN be the number of true negatives after classification. We can then define the false positive rate or fallout as FPR = FP/N = FP/FP + TN and F-measure, F1-score, or harmonic mean of TPR and SPC as HM = 2TPR/2TPR + FP + FN.

### 3.3. Quantitative and Qualitative Evaluation of Pollen Identification

The results of the pollen selection and the feature extraction phases over the described dataset are a collection of 618 pollen candidate regions divided into 12 classes according to the ground-truth. Each pollen substance is characterized by a 33-dimension feature vector. As previously mentioned, three classification techniques have been explored (RTF, MLP, and BN), also using three different cross validation schemes: *s*-fold, cross validation (*s* = 10, *s* = 5, and *s* = 2), and the full set for training and testing.  Table 6 summarizes all quantitative results. We observed that, in the toughest but more realistic classification experiment, the 2-fold cross validation (i.e., the dataset is divided into two equal parts, one used for training and the other for testing) MLP achieves the best results, which proves that this is a reasonable scheme for pollen classification. As expected, as the value of *s* increases in the *s*-fold cross validation, results improve until the classification techniques obtain full precision and recall for case of the full set.

### 3.4. Comparative Discussion

In Table 6, it is shown that the MLP classification method on the toughest classification task, known as 2-fold cross validation, produces very powerful results. In particular, MLP outperformed RTF and BN with an average precision of 96.2% against 95.5% and 93.5%, respectively. In addition, the average recall rate of MLP outperformed RTF and BN with an average of 96.1% against 95.5% and 92.9%, respectively. Finally, the F-measure average of MLP was improved to 96.1%, compared to 95.5% and 92.9% of RTF and BN, respectively.

In Table 7, the methods that have appeared in the literature for the segmentation of pollen images are presented. As can be observed, several methods do not take advantage of the color information of pollen images by instead converting the color image to its gray-scale counterpart [3, 4], and, therefore, there is missing color information. Also, the problem of overlapping pollen grains is not considered in many methods that identify borders of pollen in palynological images that contain only one pollen grain or isolated pollen grains [3, 4, 6]. The work of Dell'Anna et al. [7] is presented in an original way to identify and classify the nucleus by FT-IR and cluster features. The results obtained by Kaya et al. [6] show a precision over 90% (145 detected images from 160) when training with 440 images and evaluating with 160. In terms of the general image-processing approach, our method accounts for the shape information of pollen substances in contrast with techniques previously reported [3, 5] that only worked with circular pollen grains.

This paper proposes a scheme for segmenting different images of 12 pollen species. To filter the pollen ROI and background, we proposed a combination of MS and Otsu segmentation. After segmenting the ROI, morphological erosion is applied to the remaining subimage components to emphasize and separate the overlapping images. Finally, the separate region obtained is improved by applying boundary removal rules, dilatation, and a GVFS. Experimental results confirmed that the proposed method can efficiently segment the pollen ROI of the samples, individual and overlapping, with a 100% success rate. We compared our method using manual ground-truth. Experiments show that the performance of the proposed algorithm is close to that obtained by human segmentation.

## 4. Conclusion

This paper presents a new segmentation technique for pollen species detection and classification of individual and overlapping pollen grains. The first advantage of this new system is the detection of 100% of ROIs and the separation of overlapping pollen grains using the proposed segmentation method. The best algorithm for classification of pollen images was the MLP technique, with a precision of 96.2%, and the total time taken to build the model was 16.02 seconds on 2-fold cross validation. MLP and RTF classifiers got the lower average error of 0.002, compared to 0.027 of BN on a full set for training and evaluation. These results suggest that, among the machine learning algorithms tested, the MLP classifier has the potential to significantly improve the conventional classification methods used in medical and bioinformatic applications. Future work will involve the development of automated pollen video counters that includes segmenting pollen, feature extraction from each grain, and classification in twelve pollen categories. This may enable the production of a calendar-based disease diagnosis tool. In comparison with the results from other work, our methodology yields precision (PR), recall (RE), and F-measure (FM) results over 96% using MLP to classify the twelve pollen species with minimum hit rate losing (Tables 6 and 7). These results may serve as a platform for more complex systems able to chart pollen scheduling.

## Supplementary Material

The dataset and associated ground-truth is composed of 389 images from 12 different pollen species (Table 1). The images are formed using greater magnification and resolution (1388×1040 pixels). The experiments and resulting images are 278×208×3 pixels, as the application is in video format. To obtain the ground-truth, the contours of the pollen grains were manually identified by an expert palynologist. The Supplementary Materials for download contain the entire pollen images database (clases 1–12), the ground-truth (as binary bmp images), results images segmentation (as Matlab figures), Excel descriptors of the features of every pollen, Weka features and associated class for experiment (as ARFF Data File), and segmentation method (as Matlab interface).

## Figures and Tables

**Table 1:** Palynological classification of pollen species images dataset.

Class	Pollen common name (genus/species)	Pollen	Images
1	Huisache (*Acacia farnesiana*)	49	45
2	Alfalfa *(Medicago sativa)*	47	29
3	Nettle-leaved goosefoot *(Chenopodium murale)*	60	31
4	Chicken foot grass (*Cynodon dactylon*)	37	34
5	Grass bitter (*Helianthus ciliaris*)	43	34
6	White mulberry (*Morus alba*)	57	24
7	Pecan (*Carya illinoensis*)	32	31
8	Olive (*Olea europaea*)	28	28
9	Honey mesquite (*Prosopis glandulosa*)	86	50
10	Willow (*Salix *spp.)	64	30
11	Pepper tree (*Schinus molle*)	78	23
12	Johnson grass (*Sorghum halepense*)	34	31

	Total public pollen species images dataset	618	390

**Table 2:** Summary of descriptors.

Shape	
Area	*A* = *n* pixels

Perimeter	$P=\sqrt{{\left(x_{i}-x_{i-1}\right)}^{2}+{\left(y_{i}-y_{i-1}\right)}^{2}}$
Roundness	$R=4\pi \frac{A}{P^{2}}$
Compactness	$C=\frac{A}{P^{2}}$

First-order texture	

Average	$\mu =\frac{1}{ij}\underset{i,j}{\sum }p\left(i,j\right)$
Median	$m=L+I\left(\frac{\left(N/2\right)-F}{f}\right)$
Variance	$\sigma^{2}=\frac{1}{ij}\underset{i,j}{\sum }\left(p\left(i,j\right)-\mu \right)$
Standard deviation	$\sigma =\sqrt{\frac{1}{ij}\underset{i,j}{\sum }\left(p\left(i,j\right)-\mu \right)}$
Entropy	$S=-\underset{i,j}{\sum }p\left(i,j\right)logp\left(i,j\right)$

Second-order texture	

Contrast descriptor	$CM=\underset{i,j}{\sum }{\left|i-j\right|}^{2}c\left(i,j\right)$
Correlation	$r=\underset{i,j}{\sum }\frac{\left(i-\mu_{ci}\right)\left(j-\mu_{cj}\right)c\left(i,j\right)}{\sigma_{ci}\sigma_{cj}}$
Energy	$e=\underset{i,j}{\sum }c{\left(i,j\right)}^{2}$
Local homogeneity	$HL=\underset{i,j}{\sum }\frac{c\left(i,j\right)}{1+\left|i-j\right|}$

**Table 3:** Confusion matrixes of multilayer perceptron results.

		10-fold cross validation													5-fold cross validation													2-fold cross validation											
		Predicted pollen class													Predicted pollen class													Predicted pollen class											
		*a*	*b*	*c*	*d*	*e*	*f*	*g*	*h*	*i*	*j*	*k*	*l*		*a*	*b*	*c*	*d*	*e*	*f*	*g*	*h*	*i*	*j*	*k*	*l*		*a*	*b*	*c*	*d*	*e*	*f*	*g*	*h*	*i*	*j*	*k*	*l*
Actual pollen class	*a* = 1	45	0	0	0	0	0	0	0	1	0	0	0		44	0	0	0	0	0	0	0	1	0	0	1		45	0	0	0	0	0	0	0	1	0	0	0
	*b* = 2	0	44	0	0	1	0	0	0	1	0	0	0		0	45	0	0	0	0	0	0	1	0	0	0		0	41	0	0	0	0	0	0	5	0	0	0
	*c* = 3	0	0	55	1	0	0	0	0	0	0	0	0		0	0	55	1	0	0	0	0	0	0	0	0		0	0	55	1	0	0	0	0	0	0	0	0
	*d* = 4	0	2	2	30	0	0	0	0	3	0	0	0		0	2	1	30	0	0	0	0	4	0	0	0		0	2	1	31	0	0	0	0	3	0	0	0
	*e* = 5	0	1	0	0	41	0	0	0	0	0	0	0		0	1	0	0	41	0	0	0	0	0	0	0		0	1	0	0	41	0	0	0	0	0	0	0
	*f* = 6	0	0	0	0	0	55	0	0	0	1	0	0		0	0	0	0	0	55	0	0	0	1	0	0		0	0	0	0	0	55	0	0	0	1	0	0
	*g* = 7	0	0	0	0	0	0	31	0	0	0	0	1		0	0	0	0	0	0	32	0	0	0	0	0		0	0	0	0	0	0	32	0	0	0	0	0
	*h* = 8	0	1	0	0	0	0	0	50	0	1	0	0		0	1	0	0	0	0	0	50	0	1	0	0		0	0	0	0	0	0	0	51	0	1	0	0
	*i* = 9	0	1	0	3	0	0	0	0	74	0	2	0		0	1	0	3	0	0	0	0	74	0	2	0		0	3	0	2	0	0	0	0	73	0	2	0
	*j* = 10	0	0	0	0	0	0	0	0	0	64	0	0		0	0	0	0	0	0	0	0	0	64	0	0		0	0	0	0	0	0	0	0	0	64	0	0
	*k* = 11	0	0	1	1	0	0	0	0	3	0	68	0		0	0	1	1	0	0	0	0	2	0	69	0		0	0	1	1	0	0	0	0	1	0	70	0
	*l* = 12	0	0	0	0	0	0	0	0	0	1	0	33		1	1	0	0	0	0	0	0	0	1	0	31		0	0	0	0	0	0	1	0	0	1	0	32

**Table 4:** Confusion matrixes of random tree forests results.

		10-fold cross validation													5-fold cross validation													2-fold cross validation											
		Predicted pollen class													Predicted pollen class													Predicted pollen class											
		*a*	*b*	*c*	*d*	*e*	*f*	*g*	*h*	*i*	*j*	*k*	*l*		*a*	*b*	*c*	*d*	*e*	*f*	*g*	*h*	*i*	*j*	*k*	*l*		*a*	*b*	*c*	*d*	*e*	*f*	*g*	*h*	*i*	*j*	*k*	*l*
Actual pollen class	*a* = 1	46	0	0	0	0	0	0	0	0	0	0	0		45	0	0	0	0	0	0	0	0	0	1	0		45	0	0	0	0	0	0	0	1	0	0	0
	*b* = 2	0	45	0	0	0	0	0	0	1	0	0	0		0	44	0	0	1	0	0	0	1	0	0	0		0	41	0	0	0	0	0	0	5	0	0	0
	*c* = 3	0	0	54	1	0	0	0	0	0	0	1	0		0	0	53	1	0	0	0	0	0	0	2	0		0	0	55	1	0	0	0	0	0	0	0	0
	*d* = 4	0	1	0	36	0	0	0	0	0	0	0	0		0	2	0	32	0	1	0	0	2	0	0	0		0	2	1	31	0	0	0	0	3	0	0	0
	*e* = 5	0	0	0	0	42	0	0	0	0	0	0	0		0	0	0	0	42	0	0	0	0	0	0	0		0	1	0	0	41	0	0	0	0	0	0	0
	*f* = 6	0	0	0	0	0	56	0	0	0	0	0	0		0	0	0	1	0	55	0	0	0	0	0	0		0	0	0	0	0	55	0	0	0	1	0	0
	*g* = 7	0	0	0	0	0	0	32	0	0	0	0	0		0	0	0	0	0	0	32	0	0	0	0	0		0	0	0	0	0	0	32	0	0	0	0	0
	*h* = 8	0	0	0	0	0	0	0	52	0	0	0	0		0	0	0	0	0	0	0	52	0	0	0	0		0	0	0	0	0	0	0	51	0	1	0	0
	*i* = 9	0	3	0	2	0	0	0	0	75	0	0	0		0	4	0	3	0	0	0	0	73	0	0	0		0	3	0	2	0	0	0	0	73	0	2	0
	*j* = 10	0	0	1	0	0	0	0	0	0	63	0	0		0	0	1	0	0	0	0	0	0	63	0	0		0	0	0	0	0	0	0	0	0	64	0	0
	*k* = 11	0	0	1	0	0	0	0	0	2	0	70	0		0	0	1	0	0	0	0	0	0	0	72	0		0	0	1	1	0	0	0	0	1	0	70	0
	*l* = 12	2	0	0	0	0	0	0	0	0	1	0	31		2	0	0	0	0	0	0	0	0	1	0	31		0	0	0	0	0	0	1	0	0	1	0	32

**Table 5:** Confusion matrixes of Bayesian network results.

		10-fold cross validation													5-fold cross validation													2-fold cross validation											
		Predicted pollen class													Predicted pollen class													Predicted pollen class											
		*a*	*b*	*c*	*d*	*e*	*f*	*g*	*h*	*i*	*j*	*k*	*l*		*a*	*b*	*c*	*d*	*e*	*f*	*g*	*h*	*i*	*j*	*k*	*l*		*a*	*b*	*c*	*d*	*e*	*f*	*g*	*h*	*i*	*j*	*k*	*l*
Actual pollen class	*a* = 1	46	0	0	0	0	0	0	0	0	0	0	0		45	0	0	0	0	0	0	0	0	0	0	1		46	0	0	0	0	0	0	0	0	0	0	0
	*b* = 2	0	44	0	0	0	0	0	0	2	0	0	0		0	44	0	0	0	0	0	0	2	0	0	0		0	45	0	0	0	0	0	0	1	0	0	0
	*c* = 3	0	0	52	2	0	0	0	0	0	0	1	1		0	0	53	2	0	0	0	0	0	0	0	1		0	0	52	2	2	0	0	0	0	0	0	0
	*d* = 4	0	1	1	34	0	0	0	0	1	0	0	0		0	2	1	33	0	0	0	0	1	0	0	0		0	2	1	32	0	1	0	0	1	0	0	0
	*e* = 5	0	1	0	0	40	0	0	0	0	0	1	0		0	0	0	0	41	0	0	0	0	0	1	0		0	1	0	0	40	0	0	1	0	0	0	0
	*f* = 6	0	0	0	0	0	53	0	0	0	3	0	0		0	0	0	0	0	53	0	0	0	3	0	0		0	0	0	0	0	54	0	0	0	1	0	1
	*g* = 7	0	0	0	0	0	0	32	0	0	0	0	0		0	0	0	0	0	0	32	0	0	0	0	0		0	0	0	0	0	0	32	0	0	0	0	0
	*h* = 8	0	0	0	0	0	0	0	51	0	1	0	0		0	0	0	0	0	0	0	52	0	0	0	0		0	1	0	1	0	0	0	48	0	2	0	0
	*i* = 9	0	10	0	2	0	0	0	0	67	0	1	0		0	10	0	4	0	0	0	0	66	0	0	0		0	10	1	2	0	0	0	0	65	0	2	0
	*j* = 10	0	0	0	0	0	0	0	0	0	64	0	0		0	0	0	0	0	0	0	0	0	64	0	0		0	0	0	0	0	1	0	0	0	63	0	0
	*k* = 11	0	0	1	1	0	0	1	0	1	0	69	0		0	0	1	1	0	0	0	0	1	0	68	2		0	0	0	2	0	0	1	0	0	0	70	0
	*l* = 12	0	0	1	2	0	0	3	0	0	1	1	26		1	0	1	2	0	0	1	0	0	1	0	28		1	1	0	2	0	0	1	0	1	1	0	27

**Table 6:** Quantitative classification results.

Technique	Experiment (*s*-fold cross validation)	External quality indicators		Internal quality indicators	
		TPR	PPV	HM	FPR
MLP	*s* = 10	0.974	0.975	0.974	0.003
	*s* = 5	0.961	0.962	0.961	0.004
	*s* = 2	0.961	0.962	0.961	0.004

RTF	*s* = 10	0.955	0.955	0.955	0.005
	*s* = 5	0.955	0.956	0.955	0.005
	*s* = 2	0.955	0.955	0.955	0.005

BN	*s* = 10	0.935	0.94	0.935	0.006
	*s* = 5	0.937	0.941	0.937	0.005
	*s* = 2	0.929	0.935	0.929	0.006

BN: Bayesian network; FPR: fallout or false positive rate; HM: harmonic mean; MLP: multilayer perceptron; PPV: precision; TPR: sensitivity, recall, or true positive rate; RTF: random tree forests.

**Table 7:** Dataset comparison of the proposed method and other methods, as appeared in the literature.

Reference	Detection	Description	Classification results	Results		
Proposed	A database of 12 pollen species is generated and a MS-Otsu filter applied to separate and regroup overlapping pollen grains using morphological operations and GVFS	Shape, first- and second-order texture	Multilayer perceptron (MLP)	PR	RE	FM
			10-fold cross validation (0.9–0.1)	0.974	0.975	0.974
			5-fold cross validation (0.8–0.2)	0.961	0.962	0.961
			2-fold cross validation (0.5–0.5)	0.961	0.962	0.961

Kaya et al. [6]	Classifies 20 different pollen types obtained from the genus *Onopordum* L. (Asteraceae) by a rough set-based expert system. For each pollen grain, 30 different images were photographed (600 total)	Microscopic features: polar axis (P), equatorial axis (E), P/E, exine, intine, tectine, nexine, columella, colpus L, and colpus W	The 440 samples were used for training and the remaining 160 samples were used for testing (600 total)	The overall success of the RS method in recognition of the pollen grains PR = 90.625% (145/160 pollen samples)		

Dell'Anna et al. [7]	Discriminates and automatically classifies pollen grains from 11 different allergy-relevant species belonging to 7 different families	Fourier transform infrared (FT-IR) patterns	Applied statistical analysis unsupervised (hierarchical cluster analysis, HCA) and supervised (k-NN neighbors classifier, k-NN) learning method in the process of pollen discrimination	Obtained accuracy of 80% for the 11 species classified and 84% for 9 species		

Mitsumoto et al. [5]	Used in autofluorescence images to simplify the problem by splitting pollen into RGB channels. Assuming circularity on the particles	Particles size	Presents the relationship between the grain diameter and *B*/*R* ratio of the pollen grains	The results show that values for the pollen grains of a given species tend to cluster within a limited area of the graph (lack of quantitative results)		

Ranzato et al. [4]	Blurring the image into two bandwidths *f*_1_ and *f*_2_, to calculate Gaussian diffrences	Local jets (shape descriptor and texture information)	Bayesian classifier. Others are tried, but there is very little improvement; train-test (90%–10%) random selection… 10 times (100%–6.8%) 100 times (100%–23.2%) after based on the use of false classifications in the training data	Train-test (90%–10%) random selection… 10 times (100%–6.8%) 100 times (100%–23.2%)		

Rodriguez-Damian et al. [3]	Looks for circular grains, as most pollen grains present have this shape. Tests some edge detection techniques to find a good shape of each pollen grain	Shape: common geometrical features (CGF); statistical moments; statistical moments; Fourier descriptors Texture: Haralicks's coefficients; gray level run length statistics; gray level run length statistics	Minimum distance classifier using preselected attributes; SVM	Texture (88%); boundary features (80%); they try fusing classifiers and improving the result (89%)		
